# Optimisation of a screening platform for determining IL-6 inflammatory signalling in the senescence-associated secretory phenotype (SASP)

**DOI:** 10.1007/s10522-019-09796-4

**Published:** 2019-02-11

**Authors:** Adam Rolt, Anitha Nair, Lynne S. Cox

**Affiliations:** Department of Biochemistry, University of Oxford, South Parks Road, Oxford, OX1 3QU UK

**Keywords:** Senescence, SASP, Ageing, Aging, IL-6, HEK-Blue, mTOR, AZD8055, Inflammation, Biosensor

## Abstract

Cellular senescence has been shown to be sufficient for the development of multiple age-related pathologies. Senescent cells adopt a secretory phenotype (the SASP) which comprises a large number of pro-inflammatory cytokines, chemokines and proteases. The SASP itself is thought to be causative in many pathologies of age-related diseases, and there is growing interest in developing seno-modifying agents that can suppress the SASP. However, in order to identify new agents, it is necessary to conduct moderate to high throughput screening with robust assays for the required outcome. Here, we describe optimisation and validation of a cell-based biosensor HEK cell line for measurement of IL-6 concentrations within the range secreted into conditioned medium by primary senescent fibroblasts, adapted for a 384 well plate format suitable for library screening applications. We further show that the assay can measure changes in IL-6 secretion dependent on cell population age, and that the assay is responsive to mTOR inhibition in the senescent cells, which reduces the SASP, including IL-6. Hence, we propose that this optimised biosensor, which we term HEK-SASP, may prove of value in studies requiring robust, renewable and relatively inexpensive assays for measuring SASP factors.

**Electronic supplementary material:**

The online version of this article (10.1007/s10522-019-09796-4) contains supplementary material, which is available to authorized users.

## Introduction

Cellular senescence is a program of cell cycle exit and proliferative arrest which is initiated in response to a variety of stimuli, such as oncogene activation (oncogene induced senescence, OIS) or replicative exhaustion (replicative senescence, RS) (van Deursen 2014). Senescent cells cause premature ageing in young mice (Xu et al. 2018) and accumulate in humans with age and at the sites of age-related diseases (ARDs) (Childs et al. 2015). They contribute to age-related pathologies through both loss of local cellular homeostasis, and through the senescence-associated secretory phenotype (SASP) (Coppe et al. 2010). The SASP is the characteristic secretome of senescent cells and comprises, but is not limited to, pro-inflammatory cytokines (e.g. IL-6, IL-8) and matrix degrading enzymes (e.g. MMP-1, 3, 10) that damage surrounding tissues through chronic sterile inflammation, disrupt tissue homeostasis, and induce bystander senescence in nearby cells through paracrine signalling (Nelson et al. 2012). Accordingly, removal of senescent cells in mouse models via genetic manipulation (Baker et al. 2011; Baker et al. 2016) or treatment with agents that selectively kill senescent cells (senolytics) increases life-span and health-span (Xu et al. 2018); such treatments also result in decreases in SASP factors (Wiley et al. 2018).

The pro-inflammatory cytokine IL-6 is canonically upregulated in the SASP of cells that have undergone both OIS and RS, and plays a causal role in disease pathology (Ghosh and Capell 2016). It triggers signalling through the IL-6 receptor, that is transduced by JAK kinase activation and STAT3 dimerization together with activation of MAPK/ERK and other downstream kinases, leading to transcriptional activation of pro-survival, pro-proliferative and pro-inflammatory genes (Kojima et al. 2013). IL-6 levels can therefore can act as a robust proxy for the wider SASP both in experimental studies and in clinical samples. However, it is important to bear in mind that senescent cells may represent a small minority of all cells within the tissue or organ, even in aged individuals, hence senescent cell dependent secretion of IL-6 will result in only very low overall IL-6 concentrations in the extracellular milieu. Techniques that permit measurement of SASP factors at the concentrations found in vivo are of increasing importance, given that first generation senolytics are currently being evaluated in Phase I clinical trials in humans for therapeutic intervention in age-related diseases (ClinicalTrials.gov Identifier: NCT03513016). Moreover, screening for drugs that suppress the SASP requires highly sensitive and reliable quantification of potentially very small changes in levels of SASP components. Current antibody-based assays for IL-6 determination in the SASP (e.g. ELISA (Meyer et al. 2017), Mesoscale discovery platforms (e.g. Alimbetov et al. 2016), and immunofluorescence (e.g. Georgilis et al. 2018) are expensive, non-renewable and labour intensive, requiring multiple washing steps. Alternative methods rely on measurement of levels of IL-6 mRNA by RT-PCR, but this does not necessarily reflect levels of functional IL-6 protein, as cellular regulation may impact at multiple steps between mRNA production and secretion of functional protein, including mRNA stability, translational efficiency, post-translational processing and control of intracellular transport and secretory pathways, all of which may affect concentrations of IL-6 secreted into the extracellular medium.

Clinical analysis and drug screening programmes for senolytics and senomodifiers would therefore benefit from assays that are relatively cheap, and that minimise processing steps. Moreover, assays that rely on antibody binding to IL-6 simply measure amounts of IL-6 and not its biological effects, which may be modified in the SASP by association with other factors. The MSD platform (e.g. Alimbetov et al. 2016) in particular requires the use of conditioned medium samples lacking FBS, such that senescent cells must be cultured for at least 24 h prior to assay in serum-free medium, which may alter patterns of gene expression within the cells. Hence development of assays that represent physiological responses to functional IL-6 would provide a better means for monitoring IL-6 in the SASP in both experimental and clinical samples. In order for such assays to be useful in drug screening programmes they should also be adaptable to high throughput systems including automation.

Here, we report optimisation of a commercially available cell-based reporter assay to detect physiologically relevant amounts of IL-6 signalling in the SASP in a 384-well plate format. We demonstrate that our optimised assay can measure biologically relevant changes in IL-6 in the SASP, such as the rise in IL-6 levels in the secretome of cultured primary skin fibroblasts as they approach senescence. We show that our assay compares favourably to prevailing methods such as ELISA. We demonstrate the compatibility of our assay format with moderate to high throughput platforms, making it suitable for screening of small molecule modulators of the inflammatory SASP, and further show its utility in detecting drug-induced suppression of the SASP.

## Results

### Optimisation of a cell-based biosensor for low concentrations of IL-6

**Fig. 1:**
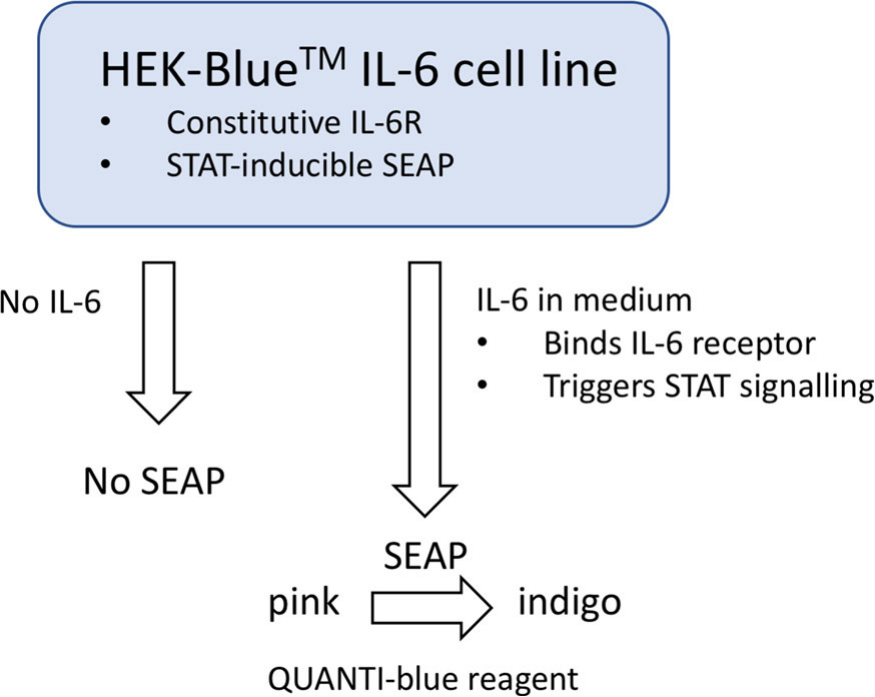
Principles of IL-6 detection by the HEK-Blue IL-6 cytokine reporter cell line. The HEK-Blue™ cell line is stably transfected with cassettes encoding both a constitutive IL-6R (interleukin 6 transmembrane receptor) and STAT3-inducible secreted embryonic alkaline phosphatase (SEAP). IL-6 (for example in senescent cell conditioned medium) binds to its cognate receptor IL-6R, triggering STAT-3 activation and STAT-3-dependent transcription of SEAP, with resultant SEAP translation and secretion into the tissue culture medium. The secreted SEAP catalyses a colour change reaction of the substrate QUANTI-blue from pink to indigo

We set out to develop a renewable, reproducible and sensitive assay suitable for measurement of IL-6 levels typically present in the secretome of senescent cells, which could be utilised in high throughput screening for agents that suppress IL-6 secretion in the SASP. We based this on a commercially available HEK cell line (HEK-Blue™ IL-6, InvivoGen) that is stably transfected with cDNA encoding the human IL-6 receptor (IL-6R) and a STAT3-responsive reporter cassette encoding secreted embryonic alkaline phosphatase (SEAP). IL-6-induced signalling resulting in secretion of SEAP can be measured simply by a colour change reaction (Fig. 1).

**Fig. 2:**
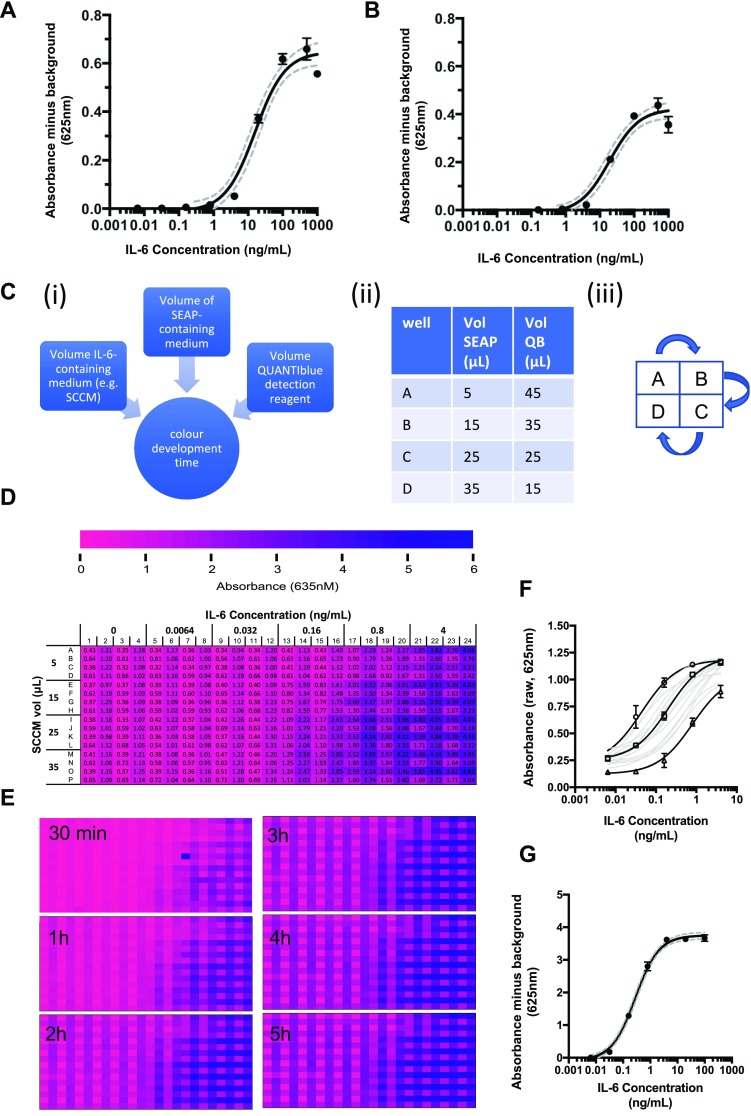
Optimisation of HEK-Blue™ IL-6 assay to detect physiological levels of IL-6. **a** Standard curve produced in a 96 well plate from a dilution series of recombinant human IL-6 (n = 2 plate replicates per concentration, with in-plate triplicates, standard deviations and 95% confidence interval shown). Conditions: 50000 HEK-Blue cells per well, 20 µL of IL-6 sample, final volume 200 µL. **b** Standard curve produced in a 384 well plate from a dilution series of recombinant human IL-6 (n = 2 plate replicates per concentration, with in-plate triplicates, standard deviations and 95% confidence intervals shown). Conditions: 12,500 cells per well, 5 µL of IL-6 sample, final volume 50 µL. **c** Schematic demonstrating optimisation protocol for HEK-SASP (i) the 4 variables tested in parallel were volume of SCCM added to HEK-Blue cells, volume of SEAP-containing medium (i.e. medium conditioned by HEK-Blue™cells), volume of QUANTI-Blue detection reagent, and colour development time for the final QB step of the assay; (ii) ratios of SEAP-containing medium to QUANTI-Blue detection reagent (QB) tested in 4 adjacent wells; (iii) pipetting of the different ratios of media in (ii) was achieved using a 96-well pipettor, with the plate shifted by one well position to the right, down or left (as shown) for each sequential pipetting reaction, leading to a quadrant format in the 384 well plate. **d** Colour coded 384 well plate with quantitative values, showing quadrant arrangement of samples. **e** Colour-coded data from incubation time course. **f** Standard curves generated from the various combinations of variables described in (**c**) and (**d**). **g** Standard curve from optimised protocol: 384 well plate, 12,500 HEK-Blue™ IL-6 cells per well, 15 µL of sample (SCCM or recombinant human IL-6) to a final volume of 50 µL. 15 µL of SEAP medium was transferred to 35 µL of QUANTI-Blue and incubated for 2 h at 37 °C in a humidified incubator at 5% CO_2_. For each curve, continuous line = mean of triplicates at each concentration within a single plate, dotted lines = 95% confidence intervals

We therefore tested the utility of this system for measurement of IL-6 at levels anticipated in conditioned medium of senescent cells grown in culture, using purified recombinant IL-6 and following the manufacturer’s protocol for the HEK-Blue assay with ~ 50,000 cells per well in 96-well plates (i.e. 4.8 million cells per plate). The procedure has two steps: (i) incubation of HEK-Blue with conditioned medium/IL-6 for 24 h, followed by (ii) assay of SEAP production in a colour development stage. As shown in Fig. 2a, the assay is capable of measuring IL-6 in the range of 5–500 ng/mL in 96 well plate format, with excellent precision (grey dotted lines represent 95% confidence intervals). While IL-6 levels as high as 50 ng/mL have been reported in conditioned medium from populations of cells where 100% are senescent (Ortiz-Montero et al. 2017), it is highly likely that the percentage of senescent cells in vivo will be at least an order of magnitude lower. Moreover, high-throughput drug screening programmes involve libraries consisting of thousands to millions of compounds, for which a 96-well plate format would not be optimal.

In order to determine whether the assay might be suitable for a 384 well plate format, we first tested scalability of the assay, simply by reducing all reagent volumes fourfold from those used in the 96 well plate format (HEK-Blue cell density was kept the same as in 96 well plates, with a fourfold reduction in number to 12,500 cells per well). As shown in Fig. 2b, scaling to 384 well plates still permitted precise measurement of IL-6 levels, in the same range of 5–200 ng/mL as was detected using the standard 96 well plate format, though signal intensity was decreased.

In order to determine conditions that would improve the sensitivity of the assay in a 384 well format, we conducted a combinatorial optimisation experiment. Four variables were adjusted across the experiment: volume of IL-6 containing medium (either recombinant IL-6 or senescent cell conditioned medium (SCCM)); volume of SEAP-containing medium harvested from HEK-Blue™ IL-6 cells after exposure to SSCM; volume of QUANTI-Blue detection reagent for SEAP measurement, and incubation times for the detection stage (Fig. 2c), using a range of concentrations of recombinant IL-6 from 0.001 to 4 ng/mL. Colour change was quantified and numerical values colour coded as shown in Fig. 2d. Varying the time allowed for colour development (detection stage) between 30 min and 5 h allowed us to determine that the majority of colour change had occurred by 2 h (Fig. 2e), a logistically favourable time frame. This combinatorial approach generated 16 independent standard curves per time point (Fig. 2f), each of which represented a viable assay protocol which can be used depending on the amount of IL-6 to be detected (i.e. suitable for multiple cell lines at various population ages, both pre- and post-senescence). Of these protocols, we found optimal conditions for reliably detecting very low IL-6 levels from 0.03 to 10 ng/mL (Fig. 2g) i.e. a sensitivity far greater than that achieved using the manufacturer’s protocol in our hands (compare Fig. 2b and g), with excellent inter-plate reproducibility (see Supplementary Fig. 1). In brief, the optimised system, which we term HEK-SASP, included seeding of 12,500 HEK-Blue cells/well in 35 µL DMEM, incubation with an additional 15 µL IL-6-containing medium for 24 h to allow time for SEAP expression and secretion, harvesting of 15 µL of this SEAP-containing medium followed by 2 h incubation with 35 µL QUANTI-Blue detection reagent, with colour change quantified using a plate reader.

### Detection of IL-6 in the SASP of senescent cells

**Fig. 3:**
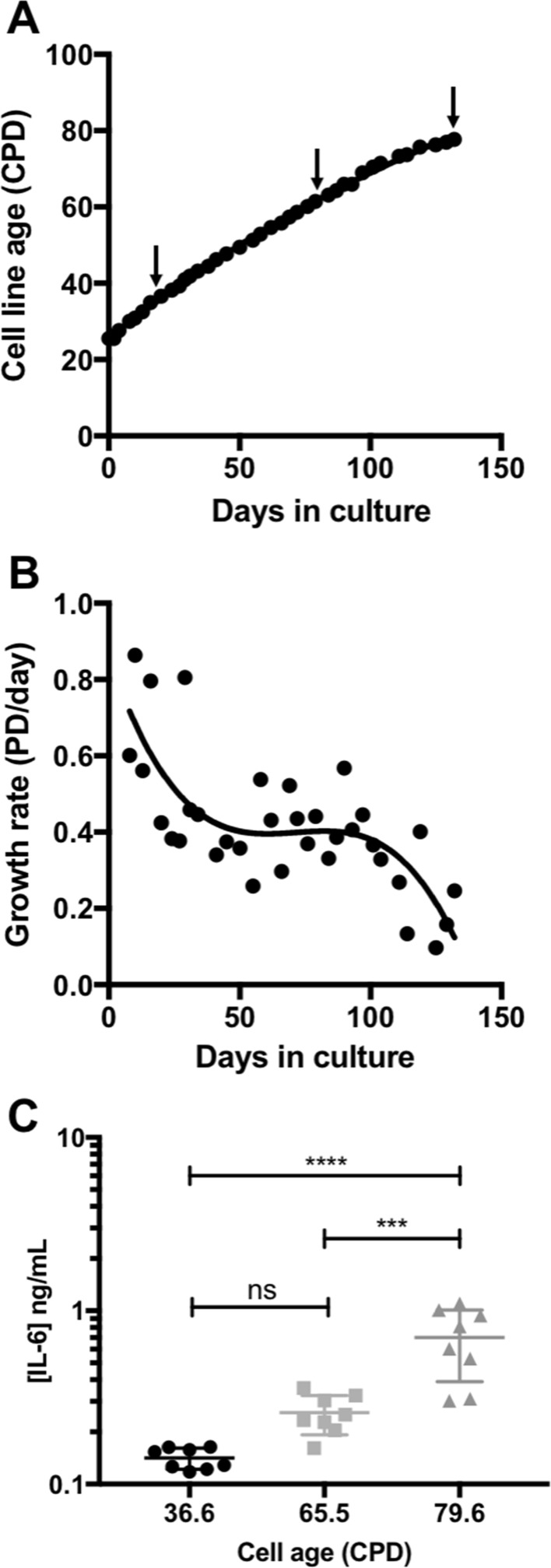
Inflammatory SASP signalling increases with cell population age. **a** HF043 fibroblasts were grown from low cumulative population doubling (CPD ~ 23) towards replicative exhaustion as described in the Methods section; samples were taken at early, mid and late CPD (arrows). **b** the rate of growth assessed as population doublings (PD) per day over time in culture. **c** IL-6 levels measured in 24 h conditioned medium from ‘young’ cells (CPD 36.6), ‘middle aged’ (CPD 65.5) or peri-senescent cells (CPD 79.6). Mean, 25th and 75% percentiles shown. One-way ANOVA, Tukeys multiple comparisons test ***p = 0.0003 ****p = < 0.0001

Having optimised the assay for IL-6 concentrations likely to be physiologically relevant using purified recombinant IL-6, we then tested whether the assay could determine IL-6 concentrations in medium conditioned by senescent cells. Primary human fibroblasts (HF043 neonatal foreskin) were cultured to replicative senescence by serial passaging from cumulative population doubling (CPD) 25 to CPD 80 (Fig. 3a), by which stage proliferation of cells within the population has dropped to less than 0.1 population doublings per day (Fig. 3b) i.e. it takes at least 10 days for the population to double in cell number. Such cells are near replicative senescence but retain limited proliferative capacity.

Conditioned medium was harvested from HF043 fibroblasts at three points along the growth trajectory (arrows in Fig. 3a) and levels of IL-6 analysed using the optimised conditions for the HEK-SASP assay. As shown in Fig. 3c, the measured IL-6 -signal increased significantly as primary skin fibroblasts progressed from young (CPD 36.6) to middle-aged (CPD 65.5) to peri-senescent states (CPD 79.6). While highly proliferative cells (CPD 36.6) uniformly secrete very little IL-6 (as measured in the HEK-SASP assay), much greater heterogeneity between samples was detected as cell populations neared senescence (Fig. 3c). This increase in heterogeneity is consistent with other reports of divergence of phenotype at late stages of cell population lifespan (Hernandez-Segura et al. 2017). Importantly, while absolute levels of detected IL-6 were low (0.1–1 ng/mL), the assay is sufficiently reproducible and sensitive to determine highly statistically significant differences between IL-6 levels secreted by cells of different population ages i.e. the assay measures IL-6 concentrations within physiological ranges. These findings of increased IL-6 detection with increasing cell population age are consistent with literature reports of SASP/IL-6 production as cells and humans age (Fagiolo et al. 1993; Wei et al. 1992; Wiley et al. 2018), suggesting that the optimised HEK-SASP assay may be useful for both lab-derived and possibly also clinical samples.

### Cross-validation of the HEK-SASP assay with IL-6 ELISA

**Fig. 4:**
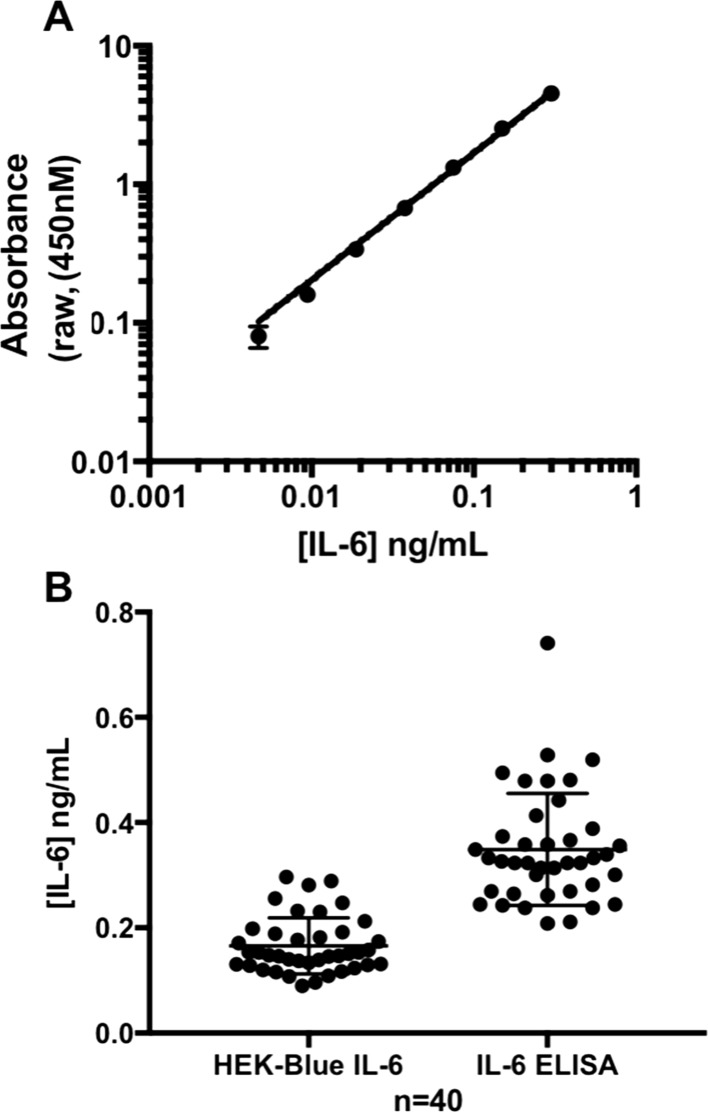
Cross-validation of the HEK-SASP assay with IL-6 ELISA. **a** ELISA standard curve generated with recombinant human IL-6. Mean ± SD **b** 40 different biological samples of 24 h senescent cell conditioned medium (SCCM) from peri-senescent HF043 fibroblasts cultured in 384 well plate format were analysed in parallel in both the HEK-SASP assay and in ELISA, standardised against recombinant human IL-6. Mean, 25th and 75% percentiles shown. (*p *< 0.0001, *n *= 40, unpaired, two-tailed *t*-test)

Having validated the HEK-SASP assay for physiological levels of IL-6. it was then important to determine how the assay compared with the current ‘gold standard’ technique of ELISA. We therefore assayed 40 samples of conditioned medium from senescent skin fibroblasts cultured in 384 well plate format in both the HEK-SASP assay and in ELISA, standardised against recombinant IL-6. While HEK-SASP gave a sigmoidal calibration curve (see Fig. 2g, above), ELISA was linear over a detection range of 0.05 to 0.8 ng/mL IL-6 (Fig. 4a). However, for biological samples (i.e. IL-6 containing SSCM rather than purified recombinant IL-6), variation within the ELISA assay was greater than that detected by the HEK-SASP assay (range ~ 0.6 ng/mL vs ~ 0.2 ng/mL respectively). The HEK-SASP assay detected levels of IL-6 varying from ~ 0.1 to 0.35 ng/mL, which were significantly lower than IL-6 concentrations detected by ELISA (0.2 to 0.78 ng/mL) (Fig. 4b, *p *< 0.0001, *n *= 40, unpaired, two-tailed *t* test). We suggest that this marked difference reflects bioavailability of IL-6 present in conditioned medium (see Discussion), which is better reported by the HEK-SASP assay.

### Potential utility of HEK-SASP assay for drug testing

Obtaining a SEAP readout in the HEK-SASP assay requires activation of a number of cellular processes including ligand-receptor binding, intracellular kinase signalling cascades, de novo gene transcription, mRNA processing, protein translation and processing, and SEAP secretion. It is also a saturable process, as shown by the sigmoidal standard curves (e.g. Fig. 2a, b, f, g). It is important to determine whether this biological complexity would rule out the use of HEK reporter cell lines in screening for SASP suppressors. mTOR inhibitors have been reported to reduce SASP signalling through suppression of IL-1a (Herranz et al. 2015; Laberge et al. 2015; Wang et al. 2017). We therefore tested the potent dual-mTORC inhibitor, AZD8055, that we have previously demonstrated reverses phenotypes of senescent cells in culture (Walters et al. 2016) for suppression of IL-6 secretion.

**Fig. 5:**
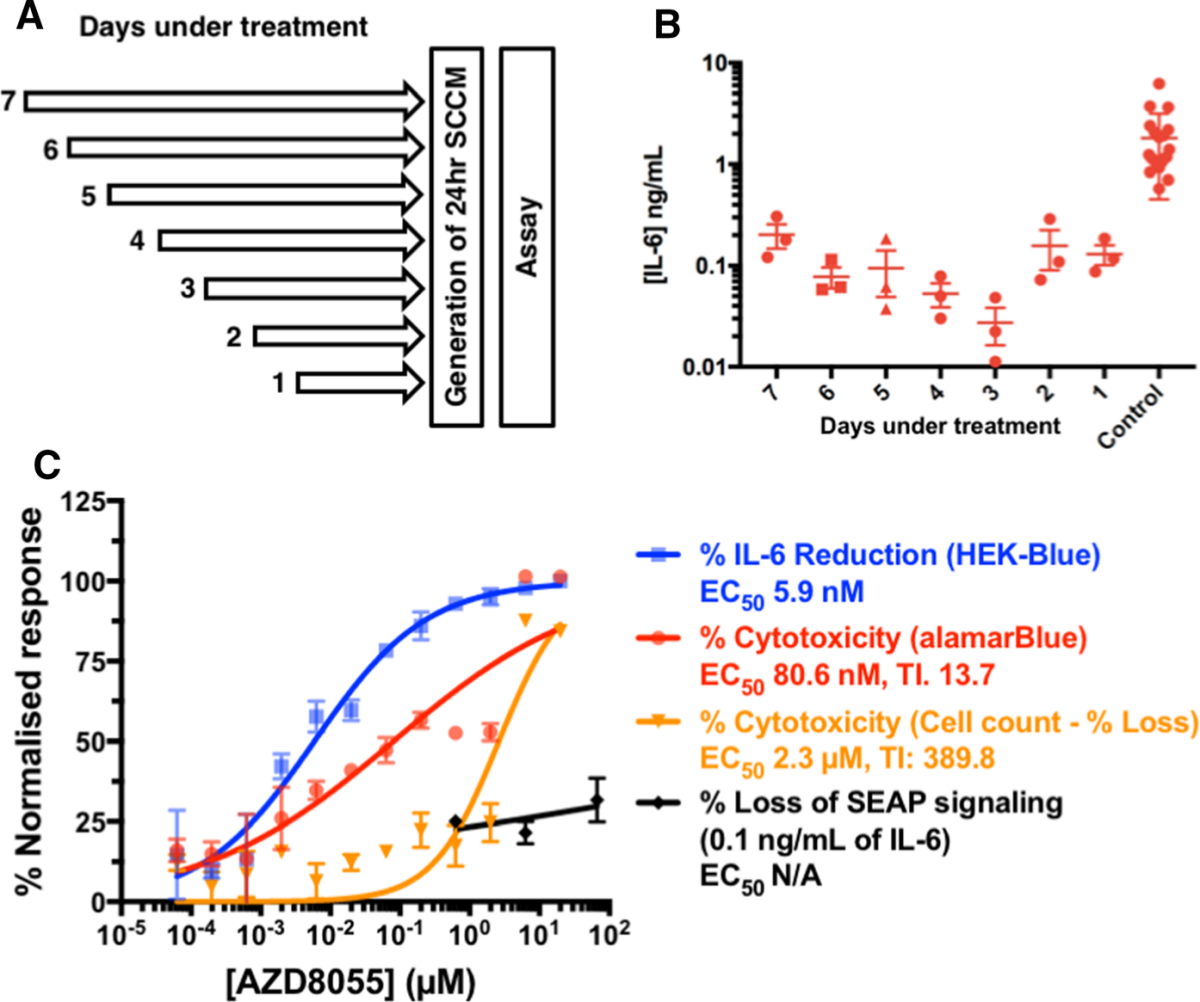
Detection of SASP suppression by pan-mTORC inhibition. **a** Schematic showing treatment regime with AZD8055; peri-senescent fibroblasts (CPD 79) were incubated with AZD8055 (70 nM) for the specified period of time (days), followed by replacement of medium (again containing AZD8055) for 24 h to generate senescent cell conditioned medium (SSCM). **b** HEK-SASP assay of conditioned medium as in (**a**). N = 3 per time point. Mean, 25th and 75% percentiles shown. **c** Peri-senescent HF043 fibroblasts were incubated with AZD8055 over a 12-point dose range for 3 days prior to analysis by HEK-SASP assay for IL-6 (blue curve); metabolic reducing capacity, a proxy of cytotoxicity by alamarBlue (red curve) or cytotoxicity assessed by direct cell counting (orange curve). HEK-Blue™ IL-6 cells were exposed to AZD8055 at 0.6–66 µM for 3 days with 0.1 ng/mL recombinant IL-6, and SEAP production measured directly using QUANTI-blue reagent (see “Methods”). Mean ± SD, n = 3 per concentration

HF043 fibroblasts at CPD 79 (peri-senescent) were incubated with AZD8055 at 70 nM for varying times over the course of 7 days (total incubation time 7 days, AZD added at 24 h intervals to parallel samples—see schematic in Fig. 5a). Suppression of IL-6 signalling was strongly detected after 24 h (1 day) of exposure of the peri-senescent cells to the mTORC inhibitor AZD8055, however a maximal reduction was obtained after 3 days of treatment (Fig. 5b). We then tested AZD8055 over a 12-point dose curve for suppression of IL-6 production by peri-senescent HF043 fibroblasts. Cells were incubated for 72 h with varying concentrations of AZD8055 and metabolic activity assessed by the vital dye alamarBlue, viability by cell counting, and IL-6 levels by the HEK-SASP assay. Data were normalised against vehicle-only controls (DMSO) for IL-6 production (DMSO levels set to 100%) and toxicity (DMSO levels set to 0%), and against hydrogen peroxide treatment as a positive control for 100% toxicity. From the data in Fig. 5c, it can be seen that AZD8055 strongly inhibits IL-6 production by peri-senescent HF043 fibroblasts (Fig. 5c, blue line) with an EC_50_ of 5.9 nM, a concentration at which there was no toxicity when measured by decrease in cell number (Fig. 5c, orange line; therapeutic index TI = 389.8), and minimally toxic by alamarBlue analysis (Fig. 5c, red line; therapeutic index TI = 13.7).

Since IL-6 detection using the HEK-SASP assay is dependent on metabolic activity of the HEK-Blue reporter line, it was therefore important to assess whether AZD8055 gave an apparent suppression of IL-6 simply because it repressed transcription/translation or other metabolic processes with the HEK cell line, consistent with the differences in curves using cell counting and alamarBlue readouts of apparent cytotoxicity. We therefore incubated the HEK-Blue™ IL-6 reporter cells directly with AZD8055 to test for reduction in SEAP production in response to a defined concentration of recombinant human IL-6 (0.1 ng/mL). We found that even at very high doses of AZD8055 (100 µM), there was minimal loss of SEAP signal (Fig. 5c, black line), suggesting that the HEK cells are relatively insensitive to AZD8055 in terms of modulation of SEAP synthesis and secretion. Hence our optimised version of this biosensor, that we term HEK-SASP, should be suitable for screening for agents that suppress the SASP, even if such agents act through transcriptional, translational or secretory pathways. Combining HEK-SASP readouts with orthogonal measurement of IL-6 levels by ELISA may even provide additional information on the biological pathways impacted during SASP suppression.

## Discussion

In recent years it has become apparent that age related diseases (ARDs) may be underpinned by several interlinked pleiotropic pathological phenotypes, particularly cellular senescence, and the accompanying pro-inflammatory SASP. In order to discover molecules that robustly modulate the SASP, it is necessary to develop screening platforms that capture the phenotypic function in pathological systems as opposed to quantifying intermediates in those pathways (e.g. measuring inflammatory signalling vs. quantifying IL-6). In this manner, any molecules discovered have a higher chance of overcoming redundancy in biological systems, and any off-target effects of the molecules that may otherwise reverse the beneficial effect are accounted for in the assay readout. It is also important to ensure that the dynamic range of such assays lies within that likely to be encountered both in the experimental system and in vivo. When studying replicative senescence, serial passaging generates cultures where upwards of 90% of the cell population has reached replicative senescence (at CPD ~ 80-90 for HF043 fibroblasts). However, the percentage of senescent cells in vivo is likely to be much lower—estimates vary according to tissue type and method of determining senescence, from < 10% in the osteoarthritic knee (Wiley et al. 2018) to > 50% in aged human skin (Lewis et al. 2011). We therefore designed the assay to be able to use conditioned medium from senescent fibroblast cultures at 5 × dilution, which gave IL-6 concentrations up to 1 ng/mL, well within the dynamic range of the calibration curves; values fivefold higher or lower would still be measurable with the system, making it of value both in laboratory-based senescence studies and in measurement of the SASP from biological/clinical samples. For biological situations in which IL-6 levels may be higher than the dynamic range of the optimised assay (e.g. SASP from oncogene-induced senescence), we suggest conducting an empirical dilution series of the samples to be assayed. Since the assay is specific for IL-6, its utility is of course restricted to systems where IL-6 is a component of the SASP. Hence a negative readout in an IL-6 assay such as this does not presuppose the total absence of SASP—for instance, senescent keratinocytes do not express IL-6, as assessed by RNAseq (Hernandez-Segura et al. 2017).

**Fig. 6:**
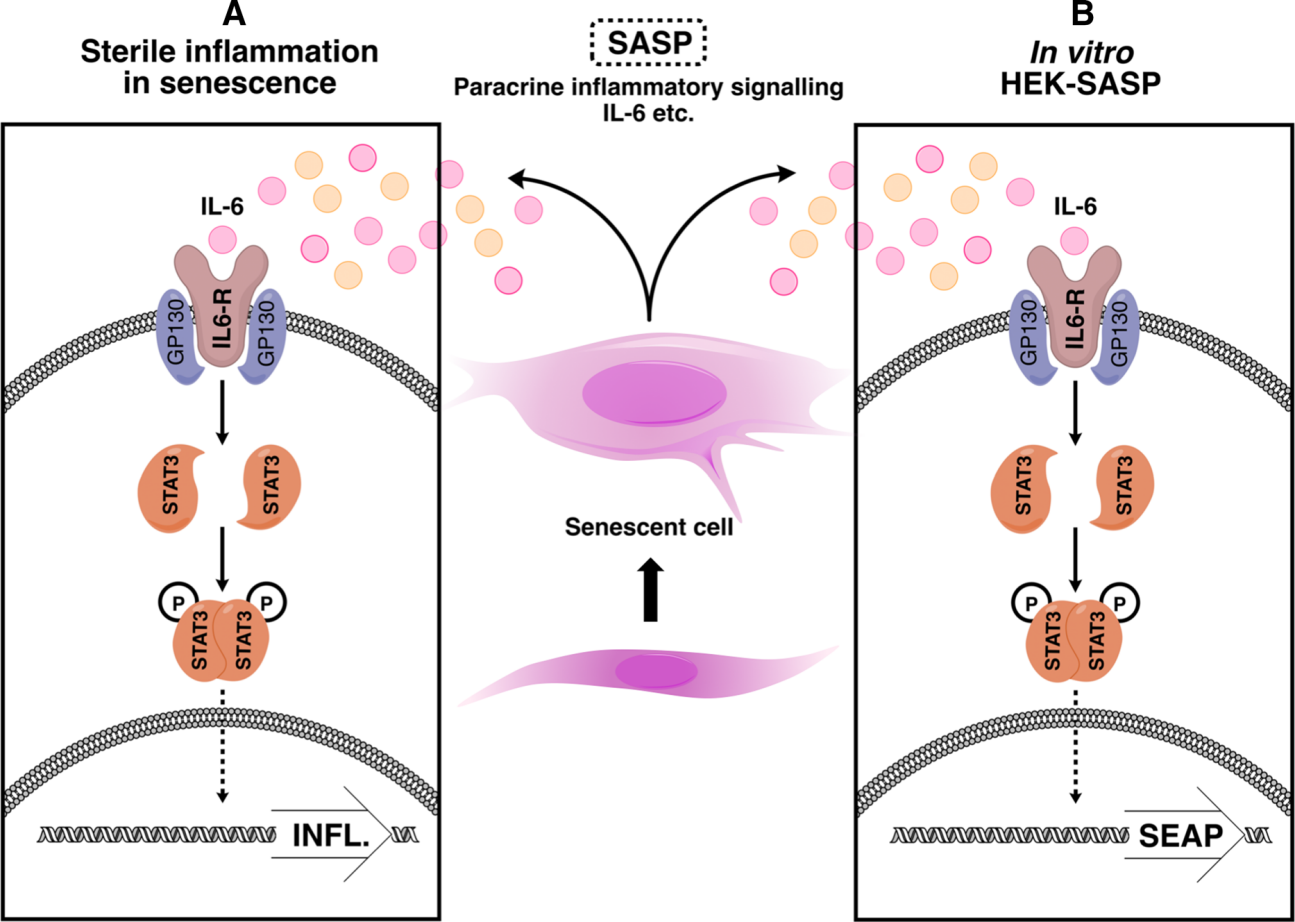
Schematic comparison between sterile inflammation in senescence versus HEK-SASP assay. **a** Schematic representation of IL-6 dependent inflammatory signalling in vivo. IL-6 binds to its cognate receptor IL-6R, leading to dimerization and activation of STAT3 and subsequent stimulation of transcription of STAT3-inducible genes, with resulting propagation of an inflammatory response. **b** Schematic representation of the HEK-SASP assay for IL-6 whereby soluble IL-6 e.g. from senescent cell conditioned medium (or recombinant IL-6) binds to the human IL-6 receptor IL-6R, stimulating activation of STAT3 which binds to and activates transcription from a stably integrated SEAP reporter construct. Secreted SEAP is detected by a colour change reaction

By positioning the STAT3 driven transcription of a SEAP reporter downstream of the human IL-6R, the HEK-Blue™ reporter cell line acts as a functional proxy for pathological paracrine inflammatory signalling in the SASP (Fig. 6). This behaviour is exemplified in Fig. 4b—the reported IL-6 signalling output from the HEK-SASP assay is significantly altered when compared to quantification of IL-6 by ELISA for identical samples. We attribute this to the increased physiological complexity of the HEK-SASP assay, which is able to reflect the presence of SASP factors in conditioned medium that lead to altered IL-6 signalling output (Fig. 6a, b). When screening for agents that suppress the SASP, we propose that a phenotypic cell-based assay, such as the HEK-SASP assay described here which encapsulates the variety of biological mechanisms by which SASP suppressors may act (IL-6 and/or IL-6R antagonism, JAK-STAT modulation, decreased mRNA transcription or processing, decreased protein translation, and/or reduced protein trafficking), is therefore less liable to false negative readouts than in vitro assays such as ELISA that measure only quantity and not activity of SASP factors. Thus, in terms of modulators of the inflammatory SASP, we propose the HEK-SASP assay provides a more physiological readout than antibody or RT-PCR based methods, and hence that it is biologically relevant to SASP determination in senescence. There is growing interest in harnessing endogenous signalling pathways to address the problem of senescent cells: a notable example is the engineering of a cell line bearing chimeric human IL-6R which, via production of a second messenger Ca^2+^ spike, activates a recombinant calcium-responsive Rho in order to permit the engineered cells to migrate towards sources of IL-6 i.e. senescent cells (Qudrat et al. 2017). As with the modified HEK-Blue™ assay, responsiveness to physiologically relevant levels of IL-6 is essential.

Having established that the HEK-SASP assay could measure inflammatory signalling in vitro at IL-6 concentrations relevant to those found in the SASP, we were able to demonstrate that inflammatory signalling increases with cellular population age (CPD), with a highly significant increase in detected IL-6 as cells near senescence (Fig. 3c). The bulk of previous reports on the SASP utilise rapid induction of senescence either through DNA damage (Rodier et al. 2009) or oncogene activation (Hoare and Narita 2017). Our data show a gradual accumulation of IL-6, and by inference the SASP, within cultured primary cell populations as they age, indicative of the stochastic but progressive nature of the onset of replicative senescence i.e. more cells in the total population become senescent as cells progress towards terminal population doubling.

The overall aim of this study was to determine the utility of the assay in a drug screening format. Dysregulation of mTOR signalling and autophagy is observed in multiple ARDs including neurodegenerative diseases, osteoarthritis and cardiovascular diseases (Walters and Cox 2018). mTOR inhibitors have therefore been investigated for their potential beneficial effects on ARDs. Notably, rapamycin has been shown to decrease secretion of SASP components in an irradiation model of senescence (Iglesias-Bartolome et al. 2012), consistent with the observation of increased lifespan and delayed onset of ARDs in naturally aged mice on oral administration of rapamycin (Cox and Mattison 2009; Harrison et al. 2009). AZD8055 is a potent pan-mTOR inhibitor (EC_50_ 0.8 nM for both mTORC1 and mTORC2; Chresta et al. 2010) that we found was able to reverse characteristic phenotypes of replicative senescence in cultured primary fibroblasts (Walters et al. 2016). As demonstrated in Fig. 5, the HEK-SASP assay reliably detected decreased IL-6 following treatment of peri-senescent fibroblasts with AZD8055, with concurrent low toxicity (as determined by both alamarBlue reducing capacity and cell counting) and comparatively insignificant effects on viability or responsiveness to recombinant IL-6 in the HEK-SASP assay i.e. the reduced SEAP signal genuinely reflects lower levels of IL-6 signalling in this system, rather than non-specific interference with the cell based HEK-SASP biosensor assay.

## Conclusions

Robust assays for drug development should implicitly control for biological redundancy and pleiotropic effects of the compounds of interest, especially in complex diseases such as ARDs. The commercially available HEK-Blue™ IL-6 reporter cell line has the potential to act as a mimic for in vivo responses to paracrine inflammatory SASP signalling, and so was optimised to detect physiological levels of IL-6 in a high throughput-compatible 384-well plate format. Through comparison with ELISA we have shown that concentration of IL-6 is not necessarily interchangeable with inflammatory signalling; importantly we have shown both that IL-6-dependent inflammatory signalling increases with cell population age as cells proceed towards replicative senescence, and that relatively small decreases in already low levels of IL-6 in conditioned medium, obtained by treatment with an mTOR inhibitor, can be detected by the optimised HEK-SASP assay. Thus we conclude that the optimised assay will be suitable to use in high throughput screening platforms for agents that suppress the SASP.

## Methods

### Cell culture

Human male neonatal foreskin primary fibroblasts (HF043, Dundee CELL products) were seeded at 8 × 10^3^ cells/cm^2^ in sterile filter-cap flasks (Greiner CELLSTAR) or ~ 1000 cells per well in 384 well plates and cultured in DMEM without phenol red (Gibco 31053-028/31053-044) supplemented with 10% FBS (Biosera, FB-1001/500) and 4 mM l-Glutamine (Sigma Aldrich). All cell incubation (including SASP and viability assays, below) was conducted at 37°C in a humidified incubator at 5% CO_2_. No antibiotics were used; mycoplasma negative status was confirmed by regular testing by PCR (Biological Industries EZ PCR Mycoplasma test kit, Geneflow K1-0210). Cells were monitored using an EVOS digital microscope (Life Technologies) and harvested at ∼ 80% confluency using TrypLE Express (Invitrogen, 12604021). After harvesting, cells were resuspended in DMEM with FBS and 20 µL of a homogenous suspension was counted using a Cellometer T4 (Nexelcom); both cell number and cell diameter in suspension were recorded. Population doublings (PD) were calculated using the formula:

### PD = log_10_(total cells harvested/total cells seeded)/log_10_2

Cumulative population doublings represent the running sum of the population doublings at every passage, indicative of cell line age. HEK-Blue IL-6 reporter cell line (InvivoGen, Cat. hkb-hil6) was cultured under identical conditions, with the addition of the HEK-Blue selection antibiotics for maintenance of the transgene (InvivoGen, Cat. hb-sel), according to the manufacturer’s instructions. Conditioned medium was obtained from HF043 fibroblasts by replacing the cell culture medium with fresh supplemented DMEM (where relevant, containing the same amount of the compound of interest) and incubating for 24 h.

### Cell-based reporter assay

Cell-based IL-6 measurement was initially carried out according to the manufacturer’s protocol (InvivoGen). Briefly, HEK-Blue cells were seeded at 50,000 cells per well into black clear-bottomed 96 well plates in 180 µL DMEM with 10% FBS, then incubated for 24 h with 20 µL of IL-6 sample or control. 20 µL of the cell culture medium was then transferred to wells of a 96-well plate containing 180 µL of 1 × QUANTI-Blue detection medium (InvivoGen, Cat. rep-qb1), and incubated for 1–3 h. Colour change indicative of secreted embryonic alkaline phosphatase (SEAP) levels was determined using a PHERAstar FS plate reader (BMG Labtech) at 620–655 nm, with MARS data processing software. Modifications to this standard assay are described within the Results section.

### IL-6 ELISA

Human IL-6 ELISA kit (Cat. 55522) and TMB substrate set (Cat. 555214) were purchased from BD Biosciences and used according to manufacturer’s instructions. Conditioned medium samples from senescent HF043 cells in a 384 well plate at ~ 1000 cells per well were diluted fivefold in DMEM-FBS prior to assay.

### Cell viability

Culture medium was removed from HF043 cells grown in 384 well plate at ~ 1000 cells per well, and replaced with 40 µL of a 1 × solution of alamarBlue cell viability reagent (ThermoFisher, Cat. DAL1100), with incubation for 2 h. Fluorescence at λ_ex_560/λ_em_590 nm was measured using a PHERAstar FS plate reader (BMG Labtech), with values normalised to negative control (0.2% DMSO) and positive control (100 µM H_2_O_2_). Cell counting was conducted using CellProfiler 3.0 cell image analysis software (Carpenter et al. 2006).

### Statistical analysis

Curve fitting ([Agonist] vs. Normalized response—Variable Slope), interpolation to standard curves, and EC_50_ determination was performed in GraphPad Prism v.7.0. Figures were created in GraphPad Prism v.7.0, Microsoft PowerPoint v.16.16.2, and ChemDraw Professional 17.1. Calculations of statistical significance (Student’s t-test (two-tailed), or one-way ANOVA) were calculated in GraphPad Prism.

### Compounds

AZD8055 was purchased from Enzo Life Sciences (ENZ-CHM193, reconstituted in DMSO and stored as stock solutions at 10 mM at − 20 °C in the dark until required.

## Electronic supplementary material

Below is the link to the electronic supplementary material.

Supplementary Fig. 1. Inter-plate reproducibility of HEK-SASP assay. Standard curves from 6 separate 384-well plates as typical examples of inter-plate reproducibility in a drug screening environment. IL-6 signalling was assessed following the optimised HEK-SASP protocol described in the text. Continuous line = mean of triplicates in each plate, dotted lines = 95% confidence intervals. Supplementary material 1 (TIFF 14660 kb)
