# Correction to: Optimisation of a screening platform for determining IL-6 inflammatory signalling in the senescence-associated secretory phenotype (SASP)

**DOI:** 10.1007/s10522-019-09803-8

**Published:** 2019-03-06

**Authors:** Adam Rolt, Anitha Nair, Lynne S. Cox

**Affiliations:** Department of Biochemistry, University of Oxford, South Parks Road, Oxford, OX1 3QU UK

## Correction to: Biogerontology 10.1007/s10522-019-09796-4

In the original publication of the article, Fig. 2 was published incorrectly. The corrected Fig. 2 is given below. The original article has been corrected.

**Fig. 2:**
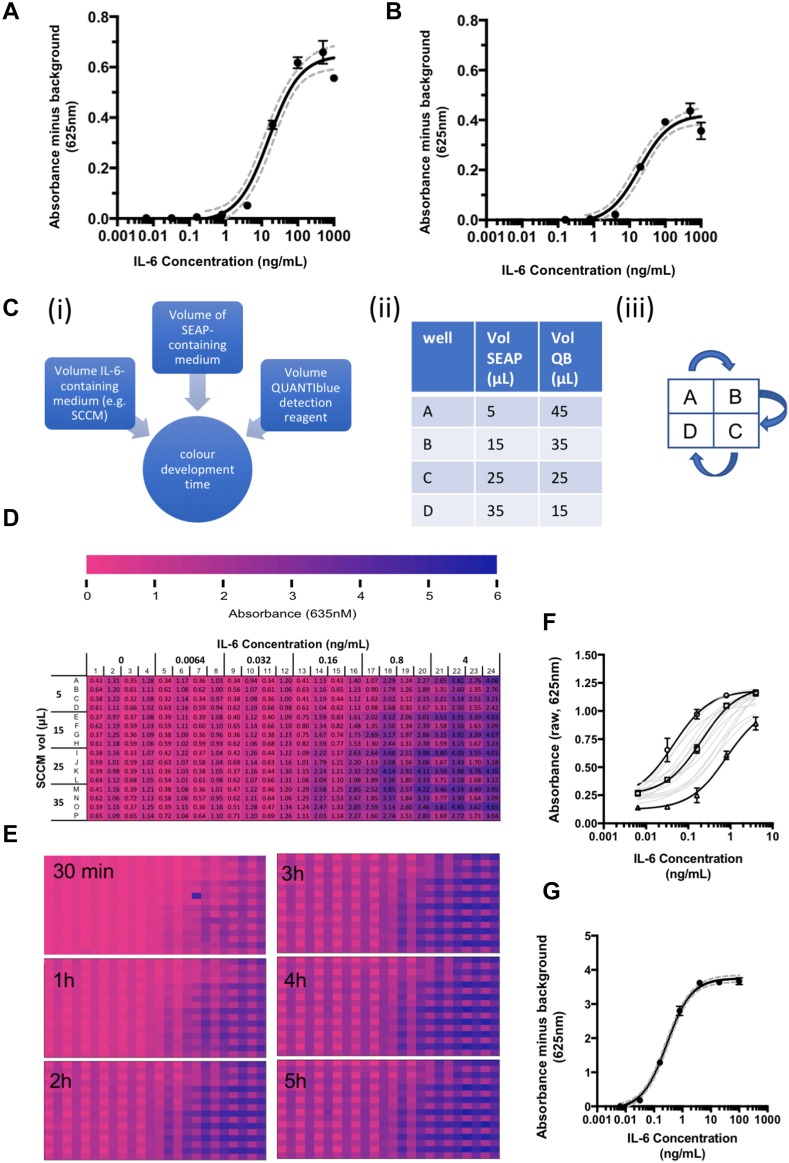
Optimisation of HEK-Blue™ IL-6 assay to detect physiological levels of IL-6. **a** Standard curve produced in a 96 well plate from a dilution series of recombinant human IL-6 (n = 2 plate replicates per concentration, with in-plate triplicates, standard deviations and 95% confidence interval shown). Conditions: 50000 HEK-Blue cells per well, 20 µL of IL-6 sample, final volume 200 µL. **b** Standard curve produced in a 384 well plate from a dilution series of recombinant human IL-6 (n = 2 plate replicates per concentration, with in-plate triplicates, standard deviations and 95% confidence intervals shown). Conditions: 12,500 cells per well, 5 µL of IL-6 sample, final volume 50 µL. **c** Schematic demonstrating optimisation protocol for HEK-SASP (i) the 4 variables tested in parallel were volume of SCCM added to HEK-Blue cells, volume of SEAPcontaining medium (i.e. medium conditioned by HEK-BlueTMcells), volume of QUANTI-Blue detection reagent, and colour development time for the final QB step of the assay; (ii) ratios of SEAP-containing medium to QUANTI-Blue detection reagent (QB) tested in 4 adjacent wells; (iii) pipetting of the different ratios of media in (ii) was achieved using a 96-well pipettor, with the plate shifted by one well position to the right, down or left (as shown) for each sequential pipetting reaction, leading to a quadrant format in the 384 well plate. **d** Colour coded 384 well plate with quantitative values, showing quadrant arrangement of samples. **e** Colour-coded data from incubation time course. **f** Standard curves generated from the various combinations of variables described in (**c**) and (**d**). **g** Standard curve from optimised protocol: 384 well plate, 12,500 HEKBlue™ IL-6 cells per well, 15 µL of sample (SCCM or recombinant human IL-6) to a final volume of 50 µL. 15 µL of SEAP medium was transferred to 35 µL of QUANTI-Blue and incubated for 2 h at 37 °C in a humidified incubator at 5% CO_2_. For each curve, continuous line = mean of triplicates at each concentration within a single plate, dotted lines = 95% confidence intervals

