# Supplementary figures and images for: Optimisation of a screening platform for determining IL-6 inflammatory signalling in the senescence-associated secretory phenotype (SASP)
Source: Biogerontology. 2019 Feb 11;20(3):359–71. doi: 10.1007/s10522-019-09796-4 (PMC6535418; doi:10.1007/s10522-019-09796-4)

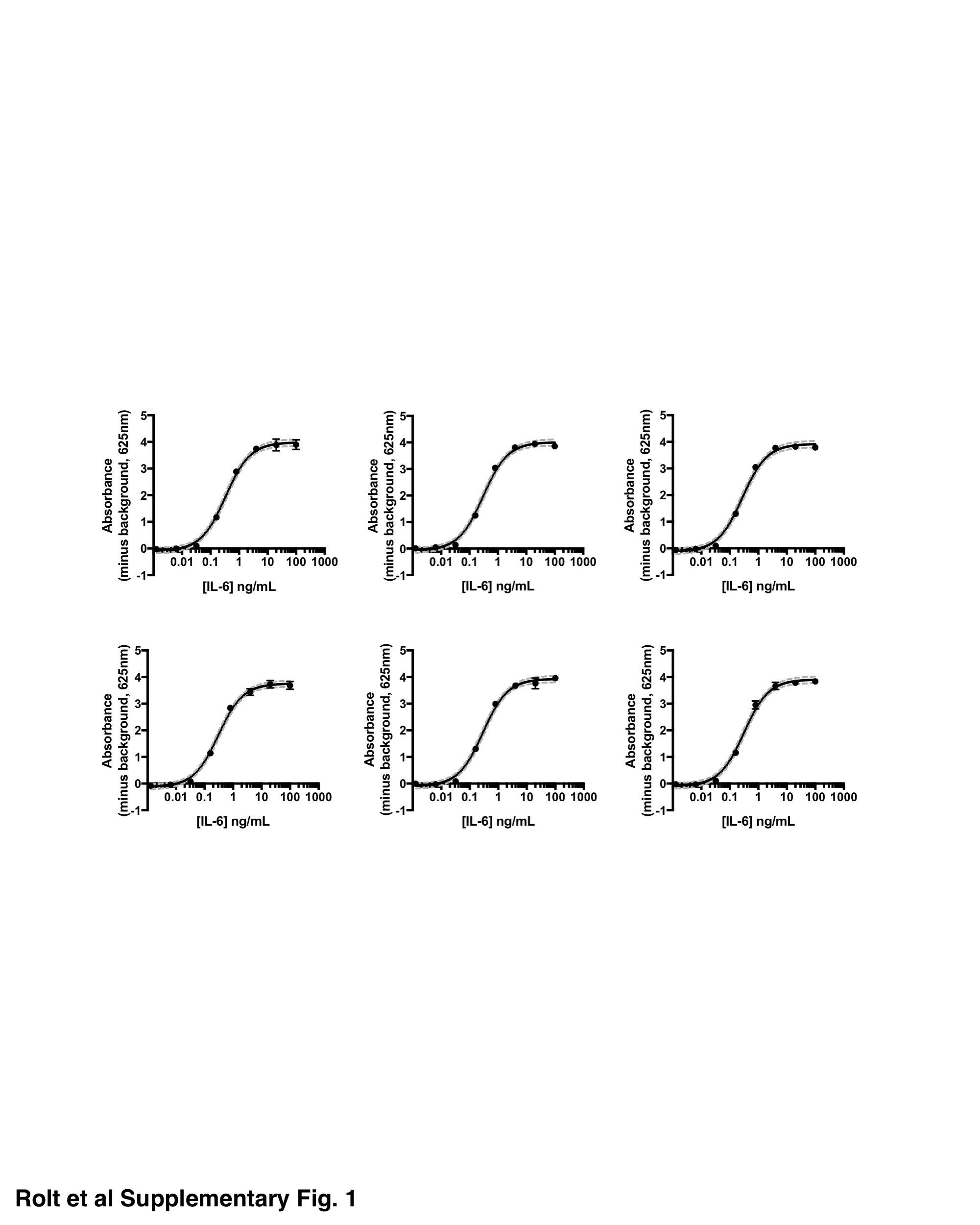

Supplement: Supplementary file 1 — Supplementary Fig. 1. Inter-plate reproducibility of HEK-SASP assay. Standard curves from 6 separate 384-well plates as typical examples of inter-plate reproducibility in a drug screening environment. IL-6 signalling was assessed following the optimised HEK-SASP protocol described in the text. Continuous line = mean of triplicates in each plate, dotted lines = 95% confidence intervals. Supplementary material 1 (TIFF 14660 kb) [file 10522_2019_9796_MOESM1_ESM.tiff]
